# Molecular search by NMR spectrum based on evaluation of matching between spectrum and molecule

**DOI:** 10.1038/s41598-021-00488-z

**Published:** 2021-10-25

**Authors:** Youngchun Kwon, Dongseon Lee, Youn-Suk Choi, Seokho Kang

**Affiliations:** 1 Samsung Advanced Institute of Technology, Samsung Electronics Co. Ltd., Yeongtong-gu, Suwon, 16678 Republic of Korea; 2 Department of Computer Science and Engineering, Seoul National University, Gwanak-gu, Seoul, 08826 Republic of Korea; 3 Department of Industrial Engineering, Sungkyunkwan University, Jangan-gu, Suwon, 16419 Republic of Korea

**Keywords:** Analytical chemistry, Cheminformatics

## Abstract

Inferring molecular structures from experimentally measured nuclear magnetic resonance (NMR) spectra is an important task in many chemistry applications. Herein, we present a novel method implementing an automated molecular search by NMR spectrum. Given a query spectrum and a pool of candidate molecules, the matching score of each candidate molecule with respect to the query spectrum is evaluated by introducing a molecule-to-spectrum estimation procedure. The candidate molecule with the highest matching score is selected. This procedure does not require any prior knowledge of the corresponding molecular structure nor laborious manual efforts by chemists. We demonstrate the effectiveness of the proposed method on molecular search using ^13^C NMR spectra.

## Introduction

In chemistry, nuclear magnetic resonance (NMR) spectroscopy is an important tool for the elucidation of chemical structures. Given an experimentally measured NMR spectrum, we analyze the resonance frequencies at which the peaks occur, called chemical shifts. Chemical shifts reflect the structural properties around spin-active atoms in the corresponding molecule, the use of which facilitates a better understanding of the chemical structure.

Because manual interpretation of an NMR spectrum is laborious and tedious, research has been conducted on the automatic determination of chemical structures from NMR spectra. A typical implementation is molecular search by NMR spectrum. For a query NMR spectrum, we search for the molecule that seems to provide the closest spectral match from a pool of candidate molecules. There are two main approaches to evaluate the matching score between the query spectrum and each candidate molecule: chemical shift similarity and spectral similarity.

**Figure 1:**
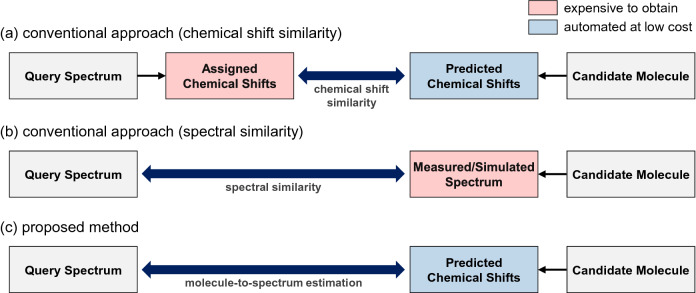
Comparison of conventional approaches and proposed method.

The first approach uses the similarity between the observed chemical shifts of the query spectrum and the predicted chemical shifts of each candidate molecule^1–4^, as illustrated in Fig. 1a. To obtain the assigned chemical shifts, the peak picking and assignment procedure is required for the raw spectrum, which often relies on the manual efforts of chemists. To obtain the predicted chemical shifts, researchers have developed various methods, including quantum chemical calculation^4–6^, search, and machine learning^1,2,9^. This approach is very efficient, but is prone to error without prior knowledge of the query spectrum, such as the chemical formula of the matching molecule. In addition, it is difficult to accurately extract chemical shifts from highly noisy spectra and complex molecules.

The second approach compares the query spectrum with the measured/simulated spectrum of each candidate molecule, as illustrated in Fig. 1b. The spectrum of the candidate molecule can be experimentally measured using NMR spectroscopy or simulated via quantum chemical calculation^10^. Various spectral similarity measures then become available for use^11–13^, which directly operate on raw spectra without an explicit annotation of the chemical shifts. This approach requires securing the spectra of all candidate molecules, which is difficult in practice. Most public NMR databases do not provide raw spectra, but only provide the assigned chemical shifts of the molecules^12^. It is impractical to directly obtain the spectra on a large scale.

In this study, we present a novel method to implement molecular search by NMR spectrum without necessitating the use of assigned chemical shifts of the query spectrum or measured/simulated spectra of candidate molecules to overcome the limitations of conventional approaches. Given a query NMR spectrum and a pool of candidate molecules with no further information, the proposed method evaluates the matching score between the query spectrum and each candidate molecule based on the molecule-to-spectrum estimation procedure, as illustrated in Fig. 1c. The candidate molecule is then evaluated to determine whether its estimated spectrum is closest to the query spectrum with minimal alignment of its predicted chemical shifts.

The main advantages of the proposed method over conventional approaches are as follows. Compared to the chemical shift similarity approach in Fig. 1a, the proposed method does not require any prior knowledge or laborious manual efforts for peak picking and assignment from the query spectrum. Compared to the spectral similarity approach in Fig. 1b, the proposed method does not require measured/simulated spectra of candidate molecules, which are expensive or otherwise difficult to obtain. These make it beneficial for implementing automated molecular search in general situations where information is limited.

## Methods

### Problem definition

The problem of molecular search by NMR spectrum is formulated as follows. Suppose a spectrum experimentally measured by NMR spectroscopy is given as a query in the form of ${\mathbf {S}}=({\mathbf {x}},{\mathbf {y}})$, where ${\mathbf {x}} = [x_1, \ldots , x_l]$ and ${\mathbf {y}} = [y_1, \ldots , y_l]$ represent the x-axis (frequency) and y-axis (intensity) of the spectrum, respectively. The corresponding molecular structure of the query spectrum ${\mathbf {S}}$ is unknown. No prior information about the molecular structure, such as the chemical formula, is available for use. We are also given a pool of candidate molecules $D=\{{\mathbf {G}}_1,\ldots ,{\mathbf {G}}_N \}$ for which experimentally measured spectra are not provided. From the candidate pool *D* with no further information, we wish to search for the best matching molecule ${\mathbf {G}}^*$ that is expected to have an NMR spectrum that is the closest match to the query spectrum ${\mathbf {S}}$.

To evaluate the matching between the query spectrum ${\mathbf {S}}$ and a candidate molecule ${\mathbf {G}}_t$, we introduce the score function that involves a molecule-to-spectrum estimation procedure. The best matching molecule ${\mathbf {G}}^*$ with the highest matching score is obtained as:

1 $$\begin{aligned} {\mathbf {G}}^* = \underset{{\mathbf {G}}_t \in D}{\arg \max } \text { } \texttt {score}({\mathbf {G}}_t;{\mathbf {S}}). \end{aligned}$$

### Molecule-to-spectrum estimation procedure

**Figure 2:**
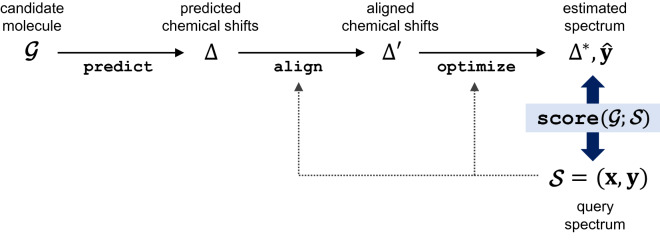
Illustration of molecule-to-spectrum estimation procedure.

Given the query spectrum ${\mathbf {S}}$ and candidate molecule ${\mathbf {G}}$, the molecule-to-spectrum estimation procedure is used to evaluate whether ${\mathbf {G}}$ has a spectrum similar to ${\mathbf {S}}$. The procedure is composed of three sequential steps, as illustrated in Fig. 2. The first step is to predict the chemical shifts of ${\mathbf {G}}$. The second step is to align the chemical shifts with ${\mathbf {S}}$. The third step is to construct a spectrum that estimates ${\mathbf {S}}$. For these three steps, we introduce the predict, align, and optimize functions.

#### From molecule to predicted chemical shifts

Given a candidate molecule ${\mathbf {G}}$, the first step is to predict the chemical shifts of its NMR-active atom, which we denote by $\Delta$, using the predict function:

2 $$\begin{aligned} \Delta =[\delta _1,\ldots ,\delta _m]=\texttt {predict}({\mathbf {G}}), \end{aligned}$$

where *m* is the number of NMR-active atoms in the molecule ${\mathbf {G}}$ and the elements of $\Delta$ are sorted in ascending order.

The success of molecular search primarily relies on the accuracy of the chemical shift prediction. Any method that provides an accurate prediction and is computationally efficient can be used in this step. In this study, we use two representative methods as the predict function:

- *Hierarchical Organization of Spherical Environments (HOSE)*^7^ A HOSE code encodes the neighborhood information around an NMR-active atom in a spherical radius. If two atoms have similar neighbors, they will have similar HOSE codes. To predict the chemical shifts of the candidate molecule ${\mathbf {G}}$, we generate HOSE codes for the NMR-active atoms in the molecule. For each NMR-active atom, we search all atoms with the same HOSE code from the NMRShiftDB2 database^14^. We then take the average shift of the atoms as the predicted chemical shift.
- *Message Passing Neural Network (MPNN)*^1^ A molecule is represented as a graph, whose nodes and edges correspond to atoms and bonds, respectively. An MPNN^15^ processes the graph representation of the molecule using multiple message passing steps to predict the chemical shifts of the NMR-active atoms in the molecule. We train the MPNN using the molecules and their annotated chemical shifts that are collected from the NMRShiftDB2 database^14^. The MPNN is then used to predict the chemical shifts of the candidate molecule ${\mathbf {G}}$.

#### Alignment of predicted chemical shifts

Given the predicted chemical shifts $\Delta$ and the query spectrum ${\mathbf {S}}$, we align the chemical shifts to ${\mathbf {S}}$ to obtain the aligned chemical shifts $\Delta '$ using the align function:

3 $$\begin{aligned} \Delta '=[\delta '_1,\ldots ,\delta '_m]=\texttt {align}(\Delta ;{\mathbf {S}}) \end{aligned}$$

The pseudocode of the align function is given below.

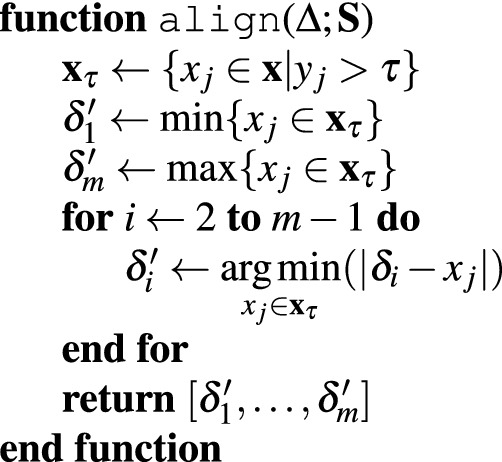



From the query spectrum ${\mathbf {S}}$, we choose the frequency values whose intensity is above a threshold $\tau$, denoted as ${\mathbf {x}}_\tau = \{{x_j \in {\mathbf {x}}| y_j > \tau }\}$. The value of $\tau$ is the minimum intensity to identify a peak. It should be chosen adequately to distinguish between peaks and noise in the spectrum, thereby ensuring that $\tau$ is greater than the smallest peak intensity and ${\mathbf {x}}_\tau$ includes all the actual chemical shifts of the spectrum. Then, each element in $\Delta$ is aligned as follows. The smallest chemical shift $\delta _1$ is aligned to the minimum value of ${\mathbf {x}}_\tau$. The largest chemical shift $\delta _m$ is aligned to the maximum value of ${\mathbf {x}}_\tau$. The other chemical shifts $\delta _i$, $i=2,\ldots ,m-1$ are aligned to their closest values in ${\mathbf {x}}_\tau$. Regarding the comparison between the candidate molecule ${\mathbf {G}}$ and query spectrum ${\mathbf {S}}$, this step allows some inaccuracies in the predicted chemical shifts $\Delta$ from HOSE or MPNN as well as in the spectrum caused by such reasons as shielding, hydrogen-bonding, and solvent effects.

#### From aligned chemical shifts to estimated spectrum

Given the aligned chemical shifts $\Delta '$ and query spectrum ${\mathbf {S}}$, this step optimizes the chemical shifts to construct an estimated spectrum of the candidate molecule ${\mathbf {G}}$ with respect to ${\mathbf {S}}$. Using the optimize function, the optimized chemical shifts $\Delta ^*$ and estimated spectrum $\hat{{\mathbf {y}}}$ are obtained as follows:

4 $$\begin{aligned} (\Delta ^*, \hat{{\mathbf {y}}})=\left( [{\delta }^*_1,\ldots ,{\delta }^*_{m}], {[}{\hat{y}}_1,\ldots ,{\hat{y}}_l]\right) =\texttt {optimize}(\Delta ';{\mathbf {S}}) \end{aligned}$$

The pseudocode of the optimize function is given below.

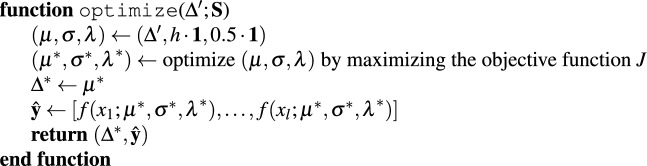



We adapt the idea of kernel density estimation^16^ to represent a spectrum as a function of chemical shifts. An estimated spectrum $\hat{{\mathbf {y}}}$ is defined by the parameters $\varvec{\mu }=[\mu _1,\ldots ,\mu _m]$, $\varvec{\sigma }=[\sigma _1,\ldots ,\sigma _m]$ and the kernel function *k* with the kernel-specific parameter $\varvec{\lambda }=[\lambda _1,\ldots ,\lambda _m]$ as:

5 $$\begin{aligned} \hat{{\mathbf {y}}} = [f(x_1;\varvec{\mu },\varvec{\sigma },\varvec{\lambda }),\ldots ,f(x_l;\varvec{\mu },\varvec{\sigma },\varvec{\lambda })];\quad f(x;\varvec{\mu },\varvec{\sigma },\varvec{\lambda }) = \sum _{i=1}^m k\left( \frac{x-\mu _i }{\sigma _i} ; \lambda _i \right) , \end{aligned}$$

where the parameters $\varvec{\mu }$ and $\varvec{\sigma }$ are associated with the chemical shifts and their peak intensities, respectively. For the kernel function *k*, we use the Gaussian-Lorentzian sum function $k(z;\lambda ) = (1-\lambda ) \exp \left( - (4 \ln 2) z^2 \right) + \lambda /(1 + {4 z^2})$ to approximate the shape of a peak in the spectrum, where $\lambda$ lies in the range of [0, 1].

The estimated spectrum $\hat{{\mathbf {y}}}$ is updated along with the parameters $(\varvec{\mu }, \varvec{\sigma },\varvec{\lambda })$ to have a similar shape to that of the query spectrum ${\mathbf {S}}$. We initialize $\varvec{\mu }$ to the values of the aligned chemical shifts $\Delta '$, $\varvec{\sigma }$ to a certain initial value *h*, and $\varvec{\lambda }$ to 0.5. Then, $(\varvec{\mu }, \varvec{\sigma },\varvec{\lambda })$ are optimized by maximizing the objective function *J* as follows:

6 $$\begin{aligned} \begin{aligned} J(\varvec{\mu }, \varvec{\sigma },\varvec{\lambda }) =&\text { cossim}({\mathbf {y}}, \hat{{\mathbf {y}}}) - {\left\| \frac{{\mathbf {y}}}{\Vert {\mathbf {y}}\Vert _1} - \frac{\hat{{\mathbf {y}}}}{\Vert \hat{{\mathbf {y}}}\Vert _1} \right\| }^2 \\&- \sum _{i=1}^{m} (\mu _i-\delta '_i)^2 - \sum _{i=1}^{m} \sigma _i^2 - \sum _{i=1}^{m-1} \max \lbrace \mu _i - \mu _{i+1} + \epsilon ,0 \rbrace ^2. \end{aligned} \end{aligned}$$

The first term corresponds to the maximization of the cosine similarity between the two spectra ${\mathbf {y}}$ and $\hat{{\mathbf {y}}}$, which is calculated as $\text {cossim}({\mathbf {y}}, \hat{{\mathbf {y}}})={{\mathbf {y}} \cdot \hat{{\mathbf {y}}}}/({\Vert {\mathbf {y}}\Vert \cdot \Vert \hat{{\mathbf {y}}}\Vert })$, to ensure they have peaks at similar frequencies. The second term is used to minimize the squared Euclidean distance between the two normalized spectra ${\mathbf {y}}/\Vert {\mathbf {y}}\Vert _1$ and $\hat{{\mathbf {y}}}/\Vert \hat{{\mathbf {y}}}\Vert _1$ to ensure they have similar overall shapes. The third and fourth terms indicate the preferences for $\mu _i$, which should be close to its initial value, and $\sigma _i$, which should have a small value. The last term indicates the penalty if $\mu _{i+1}$ is not greater than $\mu _{i}$ by a certain margin $\epsilon$, thereby encouraging the peaks to split in the estimated spectrum $\hat{{\mathbf {y}}}$. In this study, we employ the Limited-memory Broyden-Fletcher-Goldfarb-Shanno (L-BFGS) algorithm^17^ for optimization.

After performing the optimization, we obtain the optimized parameters $(\varvec{\mu }^*, \varvec{\sigma }^*)$. We regard $\varvec{\mu }^*$ as the optimized chemical shifts $\Delta ^*$. The estimated spectrum $\hat{{y}}$ is updated with $(\varvec{\mu }^*, \varvec{\sigma }^*,\varvec{\lambda }^*)$ as $[f(x_1;\varvec{\mu }^*,\varvec{\sigma }^*,\varvec{\lambda }^*),\ldots ,f(x_l;\varvec{\mu }^*,\varvec{\sigma }^*,\varvec{\lambda }^*)]$.

### Matching scoring procedure

We calculate the matching score between the query spectrum ${\mathbf {S}}=({\mathbf {x}},{{\mathbf {y}}})$ and candidate molecule ${\mathbf {G}}$ using the score function. This involves the calculation of the predict, align, and optimize functions for the molecule-to-estimation procedure. The pseudocode of the score function is as follows:

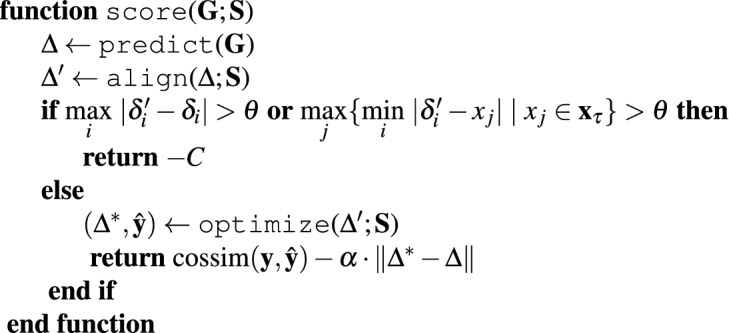



In the molecule-to-estimation procedure, the major bottleneck is the optimize function owing to its high computational cost. We do not perform the optimization if the candidate molecule ${\mathbf {G}}$ is expected to have a spectrum that is significantly different from the query spectrum ${\mathbf {S}}$. We introduce two filtering criteria. The first criterion is to abstain if the largest difference from $\Delta$ to $\Delta '$ is greater than the allowance of $\theta$, formulated as $\underset{i}{\text {max}}\text { } |\delta '_i - \delta _i | > \theta$. The second criterion is to abstain if the aligned chemical shifts $\Delta '$ fail to cover all peaks in ${\mathbf {S}}$ with a tolerance of $\theta$, formulated as $\underset{j}{\text {max}} \lbrace \underset{i}{\text {min}} \text { } |\delta '_i - x_j |\text { }|\text { } x_j \in {\mathbf {x}}_\tau \rbrace > \theta$. If either condition is met, the score function returns a matching score of $-C$:

7 $$\begin{aligned} \texttt {score}({\mathbf {G}};{\mathbf {S}}) = -C, \end{aligned}$$

where *C* is a large constant. Setting $\theta$ to a smaller value speeds up the molecular search by filtering out more candidate molecules. However, if $\theta$ is set too small, there is a risk of filtering out the actual matching molecule.

If the molecule ${\mathbf {G}}$ passes both filtering criteria, the optimized chemical shifts $\Delta ^*$ and the estimated spectrum $\hat{{y}}$ are obtained via optimization. The matching score for the query spectrum ${\mathbf {S}}$ is then calculated as follows:

8 $$\begin{aligned} \texttt {score}({\mathbf {G}};{\mathbf {S}}) = \text {cossim}({\mathbf {y}}, \hat{{\mathbf {y}}}) - \alpha \cdot \Vert \Delta ^* - \Delta \Vert , \end{aligned}$$

where the hyperparameter $\alpha$ controls the strength of the penalty for the magnitude of the alignment from $\Delta$ to $\Delta ^*$. The matching score increases if the cosine similarity between the query spectrum ${\mathbf {y}}$ and the estimated spectra $\hat{{\mathbf {y}}}$ is higher and the difference between the original predicted chemical shifts $\Delta$ and optimized chemical shifts $\Delta ^*$ is lower. The second term prevents the score from becoming spuriously high when the molecule ${\mathbf {G}}$ has many chemical shifts. The score can be negatively valued if $\Delta ^*$ is significantly different from $\Delta$.

## Results and discussion

### Dataset

We investigated the effectiveness of the proposed method on the problem of molecular search by $^{13}$C NMR spectrum. Given a $^{13}$C NMR spectrum as the query spectrum, we searched for the best matching molecule from a pool of candidate molecules.

For the query spectra, we used 36 spectra from our in-house database, which were experimentally measured using $^{13}$C NMR spectroscopy. Each spectrum was transformed into a sequence of intensity-frequency pairs with a frequency interval of 0.05ppm.

The candidate pool for the search was composed of 36 molecules that corresponded to the query spectra, another 30 molecules from the in-house database that were collected for the same purpose, and 5,000 molecules that were randomly sampled from the NMRShiftDB2 database^14^. The summary statistics of the candidate molecules used are listed in Table 1. We do not report the detailed information and query spectra of the molecules from the in-house database to comply with the confidentiality policy.

**Table 1:** Summary statistics of candidate molecules.

Source	No. molecules	No. heavy atoms per mol		No. NMR-active atoms per mol	
		Range	Avg.	Range	Avg.
In-house (query spectrum)	36	(10, 34)	20.0	(6, 32)	16.9
In-house (others)	30	(15, 61)	35.7	(12, 56)	31.7
NMRShiftDB2	5000	(3, 85)	15.2	(1, 71)	11.5

### Implementation

For the experimental investigation, we implemented the proposed method with the following configurations. For the predict function, we predicted the chemical shifts of the candidate molecules from the in-house database using the HOSE and MPNN, resulting in two different predictions per molecule, and then, we chose the prediction with the better matching score for each molecule. We used the annotated chemical shifts provided by the database itself for the candidate molecules sampled from the NMRShiftDB2 database. For the optimize function, we implemented the L-BFGS algorithm for optimization using the SciPy library^18^ in Python.

The proposed method requires the following five hyperparameters to be predetermined: $\tau , \theta , \epsilon , h$, and $\alpha$. We suggest the following guidelines for determining their values. The value of $\tau$ should be manually chosen between the highest noise and the lowest peak intensity, depending on the NMR instrument used to measure the spectrum. Choosing a proper value for *h* facilitates faster convergence of optimization. Setting the value of $\epsilon$ to be greater than 0 and smaller than the frequency interval of the query spectrum is sufficient. A smaller/larger value of $\theta$ allows more/less candidate molecules to be filtered out before optimization. The value of $\alpha$ should be chosen considering the overall scale of the chemical shifts. We note that the hyperparameters $h, \epsilon$, and $\theta$ do not significantly affect the molecular search performance but are related to the efficiency of molecular search. For the molecular search with the query spectra measured using $^{13}$C NMR spectroscopy, we used the hyperparameter settings listed in Table 2.

**Table 2:** Hyperparameter settings used for molecular search by $^{13}$C NMR spectrum.

Function	Hyperparameter	Setting	Description
Align	$\tau$	0.05	Threshold of peak intensity
Optimize	*h*	1 ppm	Initialization for $\sigma$
	$\epsilon$	0.01 ppm	Margin for peak splitting
Score	$\theta$	10 ppm	Tolerance of alignment error
	$\alpha$	0.05	Strength of penalty for alignment

Molecular search performance was evaluated in terms of the top-K accuracy. For each query spectrum, we determined whether the matching molecule was retrieved from the best K candidate molecules with the highest scores. We computed the measure with varying values of K as 1, 2, 3, 5, and 10.

### molecular search by NMR spectrum

Table 3 shows the molecular search performance for the 36 query spectra in terms of the top-K accuracy on various numbers of candidate molecules. The numbers 66 and 5,066 indicate that only the in-house database and entire molecules were respectively used to constitute the candidate pool. The proposed method achieved a considerably high top-K accuracy, indicating that it succeeded in retrieving the matching molecules from the pool for most query spectra. When the molecular search was conducted using only the in-house database as the pool, the top-1 accuracy and top-5 accuracy were 94.44% and 100%, respectively. Molecular search performance gradually decreased with the inclusion of more candidate molecules taken from NMRShiftDB2, because some of them coincidentally provided higher matching scores for some query spectra. When all 5,066 candidate molecules were considered for the search, the top-1 accuracy and top-5 accuracy decreased to 83.33% and 97.22%, respectively.

**Table 3:** Results of molecular search by ^13^C NMR spectrum.

No. candidate molecules	Top-K accuracy (%)				
	K=1	2	3	5	10
66 (In-house only)	94.44	97.22	100.00	100.00	100.00
566	94.44	97.22	100.00	100.00	100.00
1066	94.44	94.44	100.00	100.00	100.00
2066	94.44	94.44	97.22	100.00	100.00
5066 (all)	83.33	94.44	97.22	97.22	100.00

**Figure 3:**
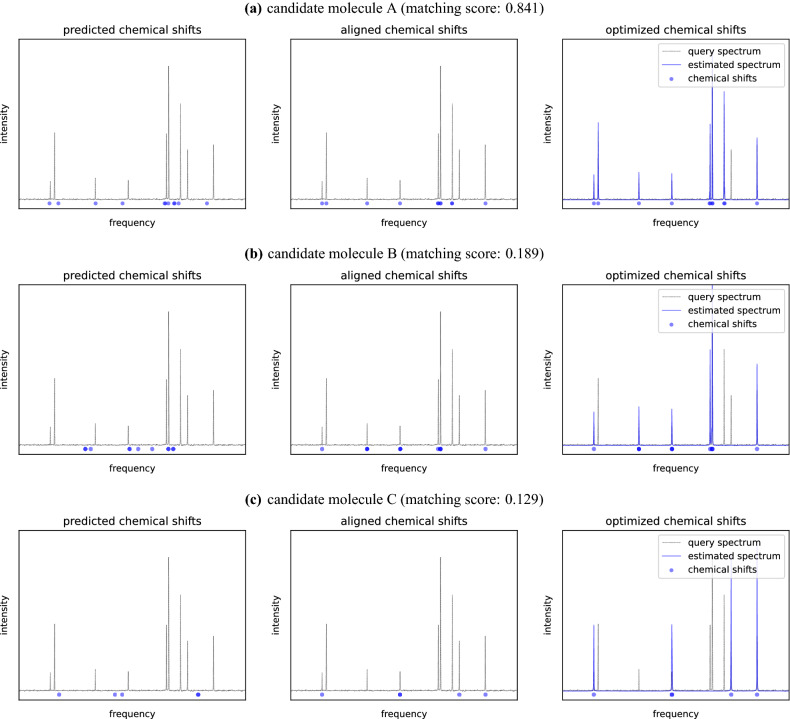
Example of molecular search by ^13^C NMR spectrum.

Figure 3 shows an example of searching from three candidate molecules given a query spectrum. We calculated the matching score of each candidate molecule with respect to the query spectrum. For candidate molecule A, its predicted chemical shifts required little alignment to match the peaks in the query spectrum. The estimated spectrum was similar to the query spectrum, and thus, its final matching score was considerably high. On the other hand, candidate molecules B and C yielded lower matching scores with respect to the query spectrum. For molecule B, the magnitude of alignment was large. For molecule C, the estimated spectrum was dissimilar to the query spectrum. Consequently, among the three molecules, we chose molecule A as the best matching molecule for the query spectrum.

**Figure 4:**
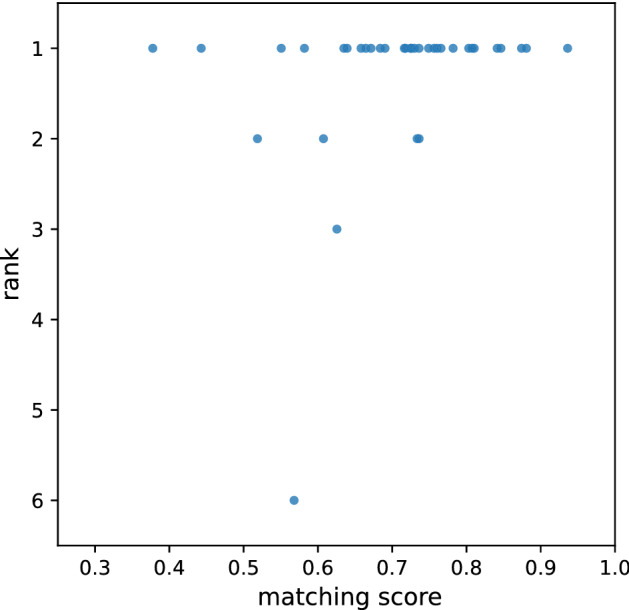
Relationship between matching score and rank for actual matching molecules of query spectra.

We investigated the relationship between the matching score and the success of the molecular search for each query spectrum. Figure 4 plots the rank among all 5,066 candidate molecules against the matching score for the actual matching molecules of the 36 query spectra. As demonstrated, when the actual matching molecule of a query spectrum yielded a high matching score, its rank among the candidate molecules was close to 1. The molecules for some query spectra yielded smaller matching scores and were subsequently ranked lower. We found that the molecular search failures were primarily caused by inaccuracies in the chemical shift prediction. Accordingly, other candidate molecules that provided moderate matching scores could take a higher rank, thereby degrading molecular search performance. We believe that molecular search performance can be improved further by enhancing the accuracy of the chemical shift prediction method used in the molecule-to-spectrum estimation procedure.

## Conclusion

In this paper, we presented a method for automated molecular search by NMR spectrum. Given a query spectrum and a pool of candidate molecules, the proposed method calculated the matching score of each candidate molecule with respect to the query spectrum by performing a molecule-to-spectrum estimation procedure. The candidate molecule with the highest matching score was retrieved by the molecular search. We demonstrated the effectiveness of the proposed method in identifying the molecules corresponding to $^{13}$C NMR spectra.

Compared with conventional approaches, the proposed method is advantageous in that it does not require any prior knowledge of the corresponding molecular structure nor laborious manual efforts by chemists to implement the molecular search. Nevertheless, incorporating prior knowledge, such as the number of NMR-active atoms and chemical formula, would be beneficial for filtering out most non-matching candidate molecules in advance, thereby further improving molecular search performance. The proposed method is versatile for any type of spectrum by adjusting the hyperparameter settings. We expect that the proposed method will prove effective in the automatic identification of molecular structures from spectra in many chemistry applications.

## Data Availability

The source code used in this study is available online at http://github.com/seokhokang/molecule_search_nmr/. For the implementation of HOSE and MPNN, we respectively used the source codes provided in https://github.com/jvansan/nmrshiftdb_predictors_app/ and https://github.com/seokhokang/nmr_mpnn_pytorch/. The NMRShiftDB2 database is publicly accessible at https://nmrshiftdb.nmr.uni-koeln.de/.
